# Prevalence of *Mycoplasma genitalium* and *Chlamydia trachomatis* in Chinese female with lower reproductive tract infection: a multicenter epidemiological survey

**DOI:** 10.1186/s12879-022-07975-2

**Published:** 2023-01-05

**Authors:** Zhan Zhang, Xiaonan Zong, Huihui Bai, Linyuan Fan, Ting Li, Zhaohui Liu

**Affiliations:** 1 The Gynecology Department of Beijing Obstetrics and Gynecology Hospital, Capital Medical University, No. 251 of Yaojiayuan Road, Chaoyang District, Beijing, China; 2 Microecological Laboratory of Beijing Obstetrics and Gynecology Hospital, Capital Medical University, No. 251 of Yaojiayuan Road, Chaoyang District, Beijing, China

**Keywords:** *Mycoplasma genitalium*, *Chlamydia trachomatis*, Lower reproductive tract infection, Prevalence, Risk factor

## Abstract

**Background:**

*Chlamydia trachomatis* and *Mycoplasma* infections have been regarded as severe challenges to public health worldwide because their potential risk of leading to serious reproductive complications. *C. trachomatis* is the most common sexually transmitted bacterial infections and the prevalence has been increasing in recent years. As a newly discovered pathogen, *Mycoplasma genitalium* has gradually been recognized as important sexually transmitted infection and even been called a “new chlamydia”. There are no official epidemiological data of *M. genitalium* in China especially in women with lower reproductive tract infection. This work aims to understand the prevalence and risk factors of *M. genitalium* and *C. trachomatis* in women with lower reproductive tract infections and to provide reference for the formulation of health policy in China.

**Methods:**

This study was conducted in the gynecological clinics of 12 hospitals geographically located in different regions in China. Women with purulent cervical secretions or abnormal vaginal microecology were included as the research group, and those with normal vaginal microecology and cervical secretions were included as the control group. A total of 2190 participants were recruited in this project including 1357 of research group and 833 of control group. All participants were required to complete questionnaires, whose vaginal discharge were collected for vaginal microecology test and cervical discharge for detection of *M. genitalium* and *C. trachomatis*.

**Results:**

The prevalence of *C. trachomatis* and *M. genitalium* were 7.1% (96/1357) and 3.8% (51/1357), respectively in research group. The prevalence of *C. trachomatis* and *M. genitalium* varied in different regions. Infection rates of *C. trachomatis* and *M. genitalium* were higher in women with abnormal vaginal microecology (*C.t P* = 0.038, *M.g P* = 0.043), especially in women with bacterial vaginosis and mixed vaginitis, of which *C. trachomatis* showed statistical differences (bacterial vaginosis, *P* = 0.035; mixed vaginitis, *P* = 0.0001) and *M. genitalium* was close to statistical differences (bacterial vaginosis, *P* = 0.057; mixed vaginitis, *P* = 0.081). Alcoholism and abnormal vaginal microecology were positively correlated with both *C. trachomatis* and *M. genitalium* infection. Increasing age, being married and multi-parity were negatively correlated with *C. trachomatis* infection. There is a positive correlation between multiple sexual partners, diversed styles of sex and *C. trachomatis* infection.

**Conclusions:**

Women with lower genital dysbiosis have an increased risk of *C. trachomatis* and *M. genitalium*. The overall prevalence of *M. genitalium* is lower than that of *C. trachomatis*, while they have similarities in the characteristics of infection. Although *M. genitalium* is not routinely screened as *C. trachomatis* in young women, attention should be paid to *M. genitalium* infection in young women with abnormal vaginal microecology or having childbearing needs.

**Supplementary Information:**

The online version contains supplementary material available at 10.1186/s12879-022-07975-2.

## Introduction

In the last decades, chlamydial infections, caused by the obligate intracellular bacteria *Chlamydia trachomatis* (*C. trachomatis*), have been the most frequently reported sexually transmitted infections (STIs) worldwide [1]. In 2020, a total of 1,579,885 cases of *C. trachomatis* infection were reported to the CDC, which corresponds to a rate of 481.3 cases per 100,000 population. Over the past 20 years, rates of chlamydial infection have been steadily increasing in both men and women. Young age is a risk factor for *C. trachomatis* infection, particularly prevalent in those younger than 25 years old. In 2020, almost two-thirds (61%) of all reported chlamydia cases are among persons aged 15–24 years [1]. Infections caused by *C. trachomatis* are often asymptomatic so routine screening is essential for the detection of infection. Its asymptomatic nature facilitates transmission between sex partners [1]. In females, the cervix is the anatomic site most commonly infected, which can manifest as cervicitis, urethritis, pelvic inflammatory disease (PID), perihepatitis even proctitis. If untreated chlamydial infections in women increase the risk of infertility and ectopic pregnancy, leading to high medical costs [2]. If a woman was infected with *C. trachomatis* during pregnancy, infants born vaginally may develop conjunctivitis or pneumonia [3]. Nucleic acid amplification testing (NAAT) is the preferred [4] strategy for both symptomatic infections and routine screening, which is recommended by Centers for Disease Control and Prevention (CDC) in America for all sexually active women younger than 25, as well as older women of high risk group based on sexual practices [5]. The epidemiological investigation of *C. trachomatis* infection in China is limited because of small or poorly performed studies. Literature has reported that prevalence of *C. trachomatis* in healthy women of childbearing age in China is up to 5.4–13.4% and 10–13% in pregnant women [6]. In females with reproductive tract infection the prevalence of *C. trachomatis* may be higher.

*C. trachomatis* infection and its complications have been known for decades, while *Mycoplasma genitalium* (*M. genitalium*), another challenging STI pathogen, has been poorly understood for a long time because of its extremely difficult cultivated nature and limited screening. *M. genitalium* was first discovered and named by Tully in 1981 from the urethral secretions of two of thirteen male patients with non-gonococcal urethritis [7]. Although the pathogenic role of *M. genitalium* in male urethritis is clear, fewer studies have been conducted among women to determine its pathogenic role in the female reproductive tract. In the World Health Organisation (WHO) global estimates of curable STIs, no data are available for *M. genitalium* probably because of the absence of sufficient epidemiological data from many parts of the world [8]. Until now *M. genitalium* has not been included in CDC routine screening. PID is an important cause of infertility and ectopic pregnancy, and *C. trachomatis* and *Neisseria gonorrhoeae* (*N. gonorrhoeae*) are recognized causes. Recently emerging data demonstrate *M. genitalium* is associated with cervicitis and PID in 10–25% of women [9]. Prevalence of *M. genitalium* among women with PID ranges from 4 to 22% in 2021 CDC STI treatment guidelines [5]. Another systematic review and meta-analysis showed the summary prevalence of *M. genitalium* in general population was 1.3% in countries with higher levels of development and 3.9% in countries with lower levels [10]. However there have been no official epidemiological data of *M. genitalium* in China not only in general population but also in women with reproductive tract infection. It would be a serious situation if we have no effective epidemiological data because of the severe reproductive damage induced by *M. genitalium.* This work aims to understand the prevalence and risk factors of *M. genitalium* and *C. trachomatis* in the female population nationwide, and to provide reference and basis for the formulation of health strategies related to *C. trachomatis* and *M. genitalium* in China. Meanwhile we also want to understand the epidemiological characteristics of *M. genitalium* differ from *C. trachomatis*.

## Methods

### Study cohort, sample collection and detection

The study was conducted in accordance with the Declaration of Helsinki and its current amendments, and the protocol was approved by the medical ethics committee of Beijing Obstetrics and Gynecology Hospital, Capital Medical University. All subjects have provided written informed consent.

**Fig. 1:**
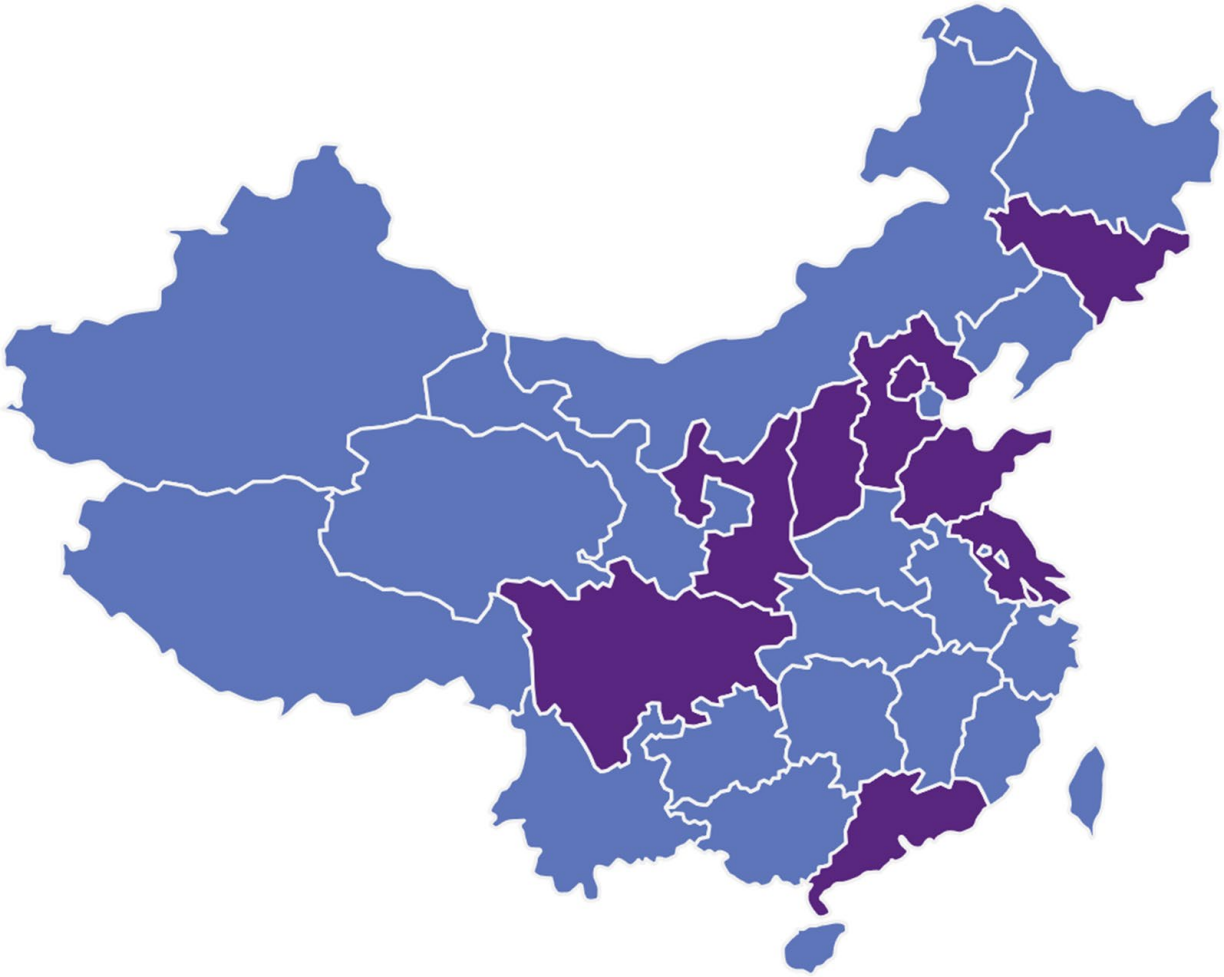
Geographical locations of twelve participating hospitals. Areas where these twelve hospitals are located are shown in purple including provinces of Jilin, Beijing, Hebei, Shanxi, Shaanxi, Shandong, Jiangsu, Shanghai, Guangdong and Chongqing etc.

This study was conducted in the gynecological clinics of 12 hospitals in China. The 12 hospitals are geographically located in different regions of China from south to north, east to west, including provinces of Jilin, Beijing, Hebei, Shanxi, Shandong, Jiangsu, Shanghai, Guangdong and Chongqing etc. (Fig. 1). Majority of participants are from urban areas. All the women recruited are heterosexual. Females with purulent cervical secretions or abnormal vaginal microecology were included as the research group, and those with normal vaginal microecology and cervical secretions were included as the control group. A total of 2190 participants were recruited in this project including 1357 of research group and 833 of control group. Inclusion criteria: women of reproductive age over than 18 years old; having sexual experience; having regular menstruation, no usage of any medications within one week; no vaginal douching, cervical treatment or sexual intercourse within 72 h [11]. Exclusion criteria: women during pregnancy, lactation or menopause and women with chronic diseases who need long-term medication.

All the recruited participants were required to complete questionnaires, including age, educational level, type of occupation, economic level, hygiene practices, number of sex partners, way of sex activity, age of first sex, number of gravidity and parity etc. Women recruited would undergo gynecological examination and vaginal microecological testing. Those with cervical purulent secretions or abnormal vaginal microecological conditions will be classified as the research group. Abnormal vaginal microecology were defined below.

Details of vaginal microecological testing: Swab of vaginal discharge was obtained from upper third of the vagina, and used for smearing, Gram staining and observing at oil lens (1000 ×) to evaluate the vaginal microecology with reference to “Evaluation of the vaginal microbiome in clinical diagnosis and management of vaginal infectious diseases” [12]. Vaginal microecology is different from vaginal microbiota. Vaginal microbiota is a general term for all the microbes in the vagina. While vaginal microecology is a comprehensive reflection of vaginal microorganisms, pH, white blood cells, vaginal cleanliness and bacterial secretion function etc. Women with the following data are considered to have a normal microecological vaginal condition [12]: pH values ranging from 3.8 to 4.5, large Gram-positive rods as predominant flora, Nugent score ≤ 3 and AV score < 3, and absence of pathogens and negative specific enzymes. The Nugent score was adopted to diagnose bacterial vaginosis (BV) (Nugent score 7–10 means BV, Nugent 4–6 means BV intermediate, Nugent 1–3 means normal). Vulvovaginal candidiasis (VVC) was indicated when hyphae or spores were discovered, and Trichomonas vaginitis (TV) was indicated when *Trichomonas* was seen under an oil lens. Clinical manifestations of aerobic vaginitis (AV) include purulent vaginal discharge, a strong inflammatory reaction and AV score ≥ 3. Flora abnormal means the dominant Lactobacillus in the vagina is replaced by other bacteria. Flora inhibition means that the bacterial species and quantity under the microscope of the secretory smear are significantly reduced, and no dominant bacteria exist. Vaginal cleanliness means the ratio of squamous epithelial cells to white blood cells in the vaginal discharge. If the number of squamous epithelial cells are more than white blood cells, we call it “vaginal cleanliness degree I”. If the number of squamous epithelial cells are similar to white blood cells, we call it degree II. If the number of white blood cells are more than squamous epithelial cells, we call it degree III. Degree III represents abnormal status.

Details of isothermal RNA amplification assay: Discharges of the cervix were collected and reserved with sterile cotton swabs and eppendorf (EP) tubes containing normal saline for *M. genitalium* and *C. trachomatis* detection. *C. trachomatis* and *M. genitalium* were tested using simultaneous amplification and testing (SAT) and isothermal RNA Amplification Assay (Rendu Biotecnology, Shanghai, China). Take N 1.5 ml clean EP tubes (N = number of samples to be tested), and add 400ul of samples to be tested. Add 100ul of nucleic acid extract into each tube (mix well before use), close the cover and shake for 30 s. Heat preservation at 60 °C for 5 min and stand at room temperature for 10 min. Put EP tube on the magnetic bead separation device, stand for 5 min, to be adsorbed on the wall of the magnetic beads, pipette suction tube cover and tube bubbles and liquid, retain the magnetic beads, after completion should be clear enough to see the magnetic beads. Add 1 ml of washing solution,wash twice, mix thoroughly and repeat the previous step. Move EP tubes away from the magnetic bead separation device, add 40 µl of expanded detection solution prepared before to each tube, shake and mix, and take 30 µl of magnetic bead suspension into a new microreaction tube. Negative and positive controls were also set up. Turn on the fluorescence detector and complete the preheating. The reaction procedure conditions of fluorescence detection instrument were set as follows: Each cycle was set at 42 °C, 1 min, for 40 cycles, and fluorescence signal was collected once every minute. Reaction system was 40 µl. Place the microreaction tube prepared above at 60 °C for 10 min, then immediately place it at 42 °C for 5 min, and preheat SAT filtrate to 42. Keep the microreaction tube at 42 °C, add 10 µl of preheated SAT enzyme solution to each tube, cover the tube immediately, shake at 1200 rpm for 15 s, mix well. Transfer the trace reaction tube to the fluorescence detection instrument quickly and start the reaction procedure immediately. Threshold setting: The threshold line was just above the highest point of the normal negative control amplification curve. Result Judgment: Decision value represents the abscissa reading at the intersection of the sample curve and the threshold line. Samples with decision value ≤ 35 are considered positive. 35 < decision value < 40 samples recommended for retesting. A sample with infinite value or 40 is considered negative.

### Statistical analysis of data

SPSS 19.0 were used for data analysis. The prevalence of *M. genitalium* and *C. trachomatis* were compared by Chi-square test or Fisher’s exact test. The risk factors of *M. genitalium* and *C. trachomatis* infection were analyzed by univariate and multivariate analysis of Logistics regression. *P* < 0.05 was statistically significant.

## Results

### Prevalence of *M. genitalium* and *C. trachomatis* in different age groups

**Fig. 2:**
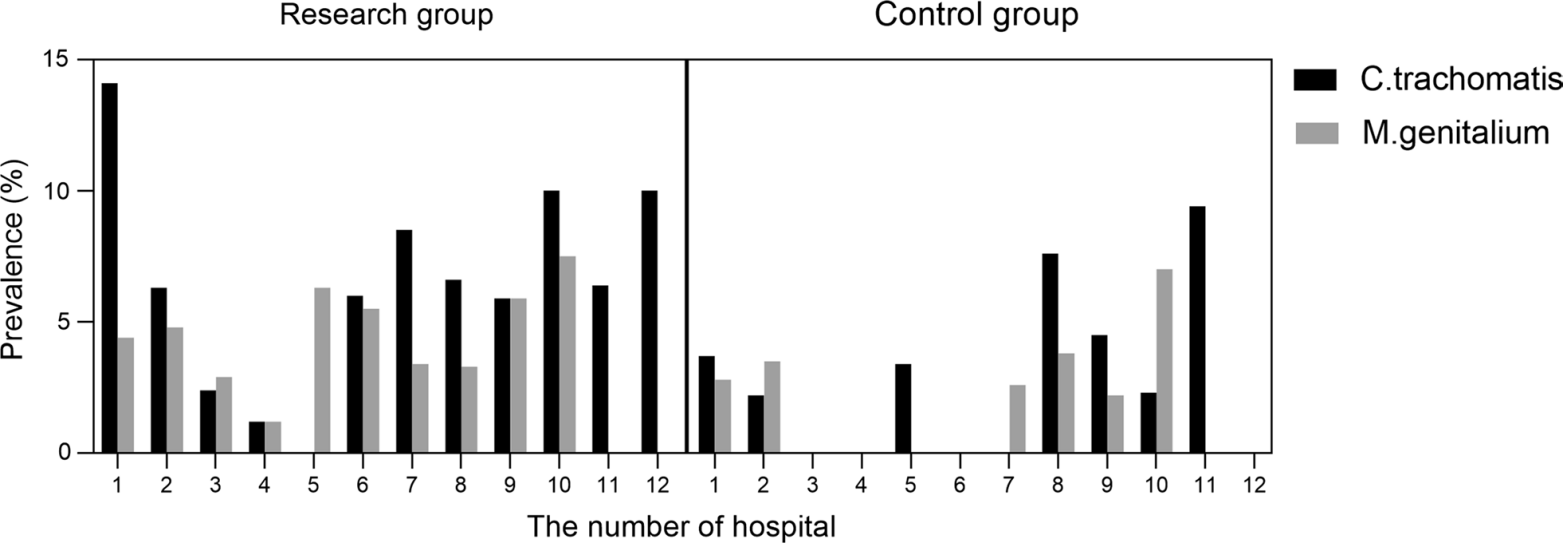
Prevalence of *C. trachomatis* and *M. genitalium* in each hospitals. Name of hospitals are listed following. 1. Peking University Shenzhen Hospital, 2. Capital Medical University Beijing Obstetrics and Gynecology Hospital, 3. The Second Affiliated Hospital of Hebei Medical University, 4. The Second Hospital of Jilin University, 5. The Second Affiliated Hospital of Shanxi Medical University, 6. Shaanxi Provincial People's Hospital, 7. Obstetrics and Gynecology Hospital of Fudan University, 8. The First Affiliated Hospital of Xi’an Jiaotong University, 9. The Second Hospital of Chongqing Medical University, 10. Nanjing Drum Tower Hospital, 11. Qilu Hospital of Shandong University, 12. Peking University First Hospital

A total of 2190 valid samples were collected in this project including 1357 of research group and 833 of control group. The 12 hospitals participated are geographically located in different regions of China (Fig. 1). Enrollment ranged from 12 to 406 at different centers, with 8 hospitals each having more than 190 participants and the other 4 hospitals having fewer than 100 participants due to a late start. We have detected 74 *M. genitalium* (+) subjects (3.4%) and 123 *C. trachomatis* (+) subjects (5.6%) totally. There were respectively 96 and 27 women with *C. trachomatis* (+) in the research group and control group with statistical significance (96/1357, 7.1% vs 27/833, 3.2%, *P* < 0.0001), while no significance has been found in the prevalence of *M. genitalium* (51/1357, 3.8% vs 23/833, 2.8%, *P* = 0.210). The prevalence varies greatly among each centers. The prevalence of *C. trachomatis* in the research group at each hospital varied from 0.0% to 14.1%, exceeding 10.0% in three hospitals, while the prevalence of *M. genitalium* ranged from 0.0% to 7.5% (Fig. 2). In contrast, the prevalence of *C. trachomatis* in the control group varied from 0.0% to 9.4%, and the prevalence of *M. genitalium* ranged from 0.0% to 7.0%, with no *M. genitalium* detected in the control group of 6 hospitals (Fig. 2).

We calculated the prevalence of *M. genitalium* and *C. trachomatis* in different age groups which were showed in Table 1. Due to the small number of people under the age of 20, we have not found *M. genitalium* infection in this part. The prevalence of *M. genitalium* has declined with increasing of age and showed bimodal distribution in 20–25 and 40–45 years old. In contrast, *C. trachomatis* infection is more common than that of *M. genitalium* and the infection rate is highest in people under 20 years old, but decreases gradually with increasing of age with no obvious bimodal distribution. The positive rate of *C. trachomatis* in the research group was significantly higher than that of the control group at the age of 20–25 years old (18.7% vs 4.3%, *P* = 0.019) while there was no statistical difference in other age groups. 45 women of unknown age were not included in the age group analysis.

**Table 1:** Prevalence of *M. genitalium* and *C. trachomatis* in different age groups

Age group	Number	*M. genitalium* prevalence			*C. trachomatis* prevalence		
		Research	Control	*P*	Research	Control	*P*
[17,20)	17	0/13 (0.0%)	0/4 (0.0%)	–	5/13 (38.5%)	1/4 (25.0%)	0.622
[20,25)	202	10/155 (6.5%)	2/47 (4.3%)	0.577	29/155 (18.7%)	2/47 (4.3%)	0.019
[25,30)	445	9/301 (3.0%)	5/144 (3.5%)	0.785	24 /301 (8.0%)	5/144 (3.5%)	0.099
[30,35)	517	15/335 (4.5%)	4/182 (2.2%)	0.188	22/335 (6.6%)	5/182 (2.7%)	0.065
[35,40)	404	4/216 (1.9%)	4/188 (2.1%)	0.843	8/216 (3.7%)	7/188 (3.7%)	0.992
[40,45)	274	8/159 (5.0%)	5/115 (4.3%)	0.793	4 /159 (2.5%)	3/115 (2.6%)	0.962
≥ 45	286	4/156 (2.6%)	3/130 (2.3%)	0.889	3/156 (1.9%)	3/130 (2.3%)	0.821
Unknown	45	1/22 (4.5%)	0/23 (0.0%)	0.301	1 /22 (4.5%)	1/23 (4.3%)	0.974
Total	2190	51/1357 (3.8%)	23/833 (2.8%)	0.210	96 /1357 (7.1%)	27/833 (3.2%)	< 0.0001

The P value was calculated by Chi-square test or Fisher’s exact probability method

### Vaginal microecology and *M. genitalium*/*C. trachomatis* infection

Prevalence of *C. trachomatis* was significantly higher in women with vaginal cleanliness over grade III (7.8% vs 3.8%, *P* < 0.0001), while prevalence of *M. genitalium* was not affected by vaginal cleanliness, which was showed in Table 2. The positive rates of *M. genitalium* and *C. trachomatis* in women with abnormal vaginal microecology were significantly higher than those with normal vaginal microecology (*M.g*, 4.2% vs 2.6%, *P* = 0.043; *C.t*, 7.1% vs 4.2%, *P* = 0.038), seen in Table 3. We made a more detailed classification of abnormal vaginal microecology and observed significantly higher *C. trachomatis* infection rates in women with mixed vaginitis and BV, while significant difference was nearly approached in *M. genitalium* infection. Infection rates of *M. genitalium* and *C. trachomatis* were slightly higher in women with other types of vaginitis, such as VVC, AV, TV, flora inhibition or flora abnormal compared to those with normal microecology, but the difference was not statistically significant. A total of 10 patients were co-infected with *M. genitalium* and *C. trachomatis*, including 5 cases with vaginal cleanliness over grade III, 1 case with BV intermediate, 1 case with BV, 1 case with VVC, 2 cases with abnormal flora, 2 cases with mixed vaginitis, 2 cases with normal microecology and 1 case with incomplete information. Among the 10 co-infected women, there were 9 persons aged 20–35 years old and 1 person aged 40–45 years old.

**Table 2:** Prevalence of *M. genitalium* and *C. trachomatis* in women of different vaginal cleanliness

Vaginal cleanliness	Number	*M. genitalium* prevalence		*P*	*C. trachomatis* prevalence		*P*
		(+)	(−)		(+)	(−)	
< III	1142	36 (3.2%)	1106 (96.8%)	0.508	43 (3.8%)	1099 (96.2%)	**< 0.0001**
≥ III	1008	37 (3.7%)	971 (96.3%)		79 (7.8%)	929 (92.2%)	
Total	2150	73 (3.4%)	23 (96.6%)		122 (5.7%)	1008 (94.3%)	

The *P* value was calculated by Chi-square test

**Table 3:** Prevalence of *M. genitalium* and *C. trachomatis* in women of vaginitis

Vaginal microbiology	Number	*M. genitalium* prevalence		*P*	*C. trachomatis* prevalence		*P*
		(+)	(−)		(+)	(−)	
Normal	1076	28 (2.6%)	1048 (97.4%)		45 (4.2%)	1031 (95.8%)	
Abnormal	1077	45 (4.2%)	1032 (95.8%)	0.043	76 (7.1%)	1001 (92.9%)	0.038
Mixed vaginitis	181	9 (5.0%)	172 (95.0%)	0.081	20 (11.1%)	161 (88.9%)	0.0001
VVC	333	12 (3.6%)	321 (96.4%)	0.337	19 (5.7%)	314 (94.3%)	0.244
AV	63	2 (3.2%)	61 (96.8%)	0.783	4 (6.4%)	59 (93.6%)	0.410
BV	244	12 (4.9%)	232 (95.1%)	0.057	18 (7.4%)	226 (92.6%)	0.035
BV intermediate	36	2 (5.6%)	34 (94.4%)	0.282	1 (2.8%)	35 (97.2%)	0.677
Flora inhibition	22	1 (4.6%)	21 (95.4%)	0.574	1 (4.6%)	21 (95.4%)	0.933
Flora abnormal	189	6 (3.2%)	183 (96.8%)	0.654	12 (6.4%)	177 (93.6%)	0.186
TV	9	1 (11.1%)	8 (88.9%)	0.115	1 (11.1%)	8 (88.9%)	0.305

The *P* value was calculated by Chi-square test or Fisher’s exact test. All *P* values were calculated by comparing with those of normal vaginal microbiology

### Risk factors for *M. genitalium* and *C. trachomatis* infection

In order to understand the risk factors of *M. genitalium* and *C. trachomatis* infection, we used Logistics regression model analysis to conduct univariate and multivariate analysis on relevant data, and calculated OR value and 95% confidence interval (CI). Tables 4 and 5, respectively show the results of univariate and multivariate analysis for *M. genitalium* and *C. trachomatis*. Univariate analysis of *M. genitalium* infection showed that alcohol abuse (OR = 3.138, 95%CI 1.308–7.529, *P* = 0.0104) and abnormal vaginal microecology (OR = 1.632, 95%CI 1.010–2.636, *P* = 0.0453) were positively correlated with *M. genitalium* infection. Multivariate analysis further confirmed that abnormal vaginal microecology (OR = 1.635, 95%CI 0.941–2.841, *P* = 0.0812) was independent risk factors for *M. genitalium* infection.

**Table 4:**
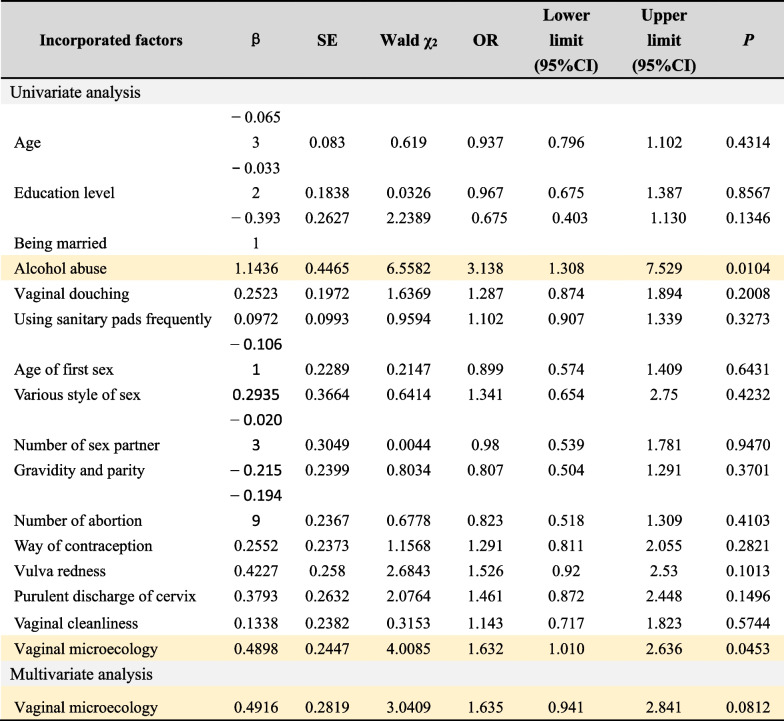
Univariate and multivariate analysis for *M. genitalium* infection

Risk factors for *M. genitalium* infection are highlighted in yellow. Value assignment of incorporated factors are detailed in the Additional file 1. “Alcohol abuse” was defined as a state of dependence on alcohol due to prolonged or repeated drinking, which has maintained for more than 12 months

**Table 5:**
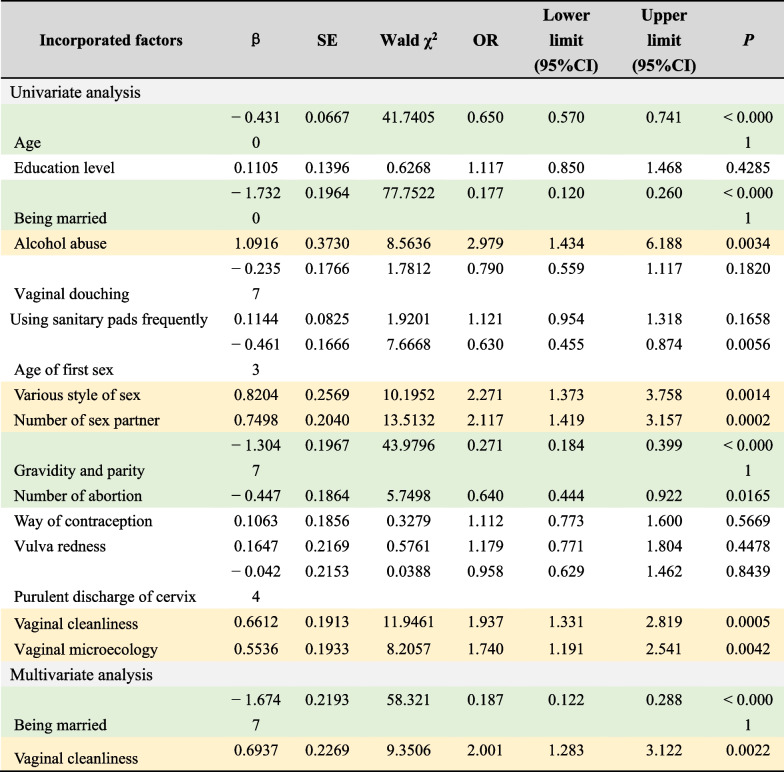
Univariate and multivariate analysis for *C. trachomatis* infection

Risk factors for *C. trachomatis* infection are highlighted in yellow, protective factors for *C. trachomatis* infection are highlighted in green. Value assignment of incorporated factors are detailed in the Additional file 1. “Alcohol abuse” was defined as a state of dependence on alcohol due to prolonged or repeated drinking, which has maintained for more than 12 months

Univariate analysis of *C. trachomatis* infection showed that increasing age (OR = 0.650, 95%CI 0.570–0.741, *P* < 0.0001), being married (OR = 0.177, 95%CI 0.120 ~ 0.260, *P* < 0.0001), gravidity and parity (OR = 0.271, 95%CI 0.184–0.399, *P* < 0.0001) and the number of abortion (OR = 0.640, 95%CI 0.444–0.922, *P* = 0.0165) was negatively correlated with *C. trachomatis* infection, while alcohol abuse (OR = 2.979, 95%CI 1.434–6.188, *P* = 0.0034), various style of sex (OR = 2.271, 95%CI 1.373–3.758, *P* = 0.0014), multiple sexual partners (OR = 2.117, 95%CI 1.419–3.157, *P* = 0.0002), poor vaginal cleanliness (OR = 1.937, 95%CI 1.331–2.819, *P* = 0.0005) and abnormal vaginal microecology (OR = 1.740, 95%CI 1.191–2.541, *P* = 0.0042) was positively correlated with *C. trachomatis* infection. Multivariate analysis showed that being married (OR = 0.187, 95%CI 0.122–0.288, *P* < 0.0001) was a protective factor and poor vaginal cleanliness (OR = 2.001, 95%CI 1.283–3.122, *P* = 0.0022) was an independent risk factor for *C. trachomatis* infection.

## Discussion

Non-viral curable STIs such as *C. trachomatis*, *N. gonorrhoeae*, and Mycoplasma infections have been regarded as serious challenges to public health throughout the world because of their potential risk of leading to a range of serious reproductive complications like PID, ectopic pregnancy and infertility [13]. *C. trachomatis* is the most common STI worldwide with an estimated 105.7 million cases in adults aged 15–49 [14]. Prevalence of STIs has been increasing in recent years, with 90 million new cases of *C. trachomatis* occurring each year, many of which are asymptomatic, and hence undiagnosed and untreated [15]. As a newly discovered pathogen of Mycoplasma, *M. genitalium* has gradually been recognized as important STIs. *M. genitalium* has even been called a “new chlamydia” [10]. However, the epidemiological investigation, pathogenisis and mechanism of *M. genitalium* have not been fully studied, especially in female population and those female at high risk of reproductive tract infection. In a systematic review [16], the incidence of *M. genitalium* was 1.07 per 100 person-years in women in very highly developed countries. Existing data showed that prevalence of *M. genitalium* are higher in patients attending sexual health clinics which is estimated to be 10–35% [17]. Among female sex workers, women visiting STI clinic, or women with cervicitis and PID, infection rates of *M. genitalium* fluctuate between 10% and 19.2% [18]. There has been no available data in China. Therefore we conducted a multi-center epidemiological survey in over ten female reproductive tract infection clinics in China, systematically investigated the infection rate of *M. genitalium* and *C. trachomatis* in women with lower reproductive tract infectious diseases, which may provide a theoretical basis for making relevant health policies. Women with purulent cervical secretions or abnormal vaginal microecology were recruited as research group. The prevalence of *C. trachomatis* and *M. genitalium* varied in different regions. The prevalence of *C. trachomatis* was significantly higher in the research group than in the control group in general (7.1% vs 3.2%). Although the prevalence of *M. genitalium* was higher in the research group as compared to the control group (3.8% vs 2.8%) statistical significance was not reached. Among women with vaginal discharge cleanliness greater than degree III, infection rates of *C. trachomatis* and *M. genitalium* were 7.8% and 3.7%, respectively, only *C. trachomatis* showed significant differences. These data gave us the illusion that *M. genitalium* infection may be less common and severe than *C. trachomatis*.

However, when we divided the included women into more detailed age groups, we could see that the positive rate of *M. genitalium* in women under the age of 20–25 could reach 6.5%, who were at a critical age of reproductive health and fertility. Women in this age group are more likely to have abnormal vaginal microbiology due to active sex. We have found the positive rates of *C. trachomatis* and *M. genitalium* were higher in women with abnormal vaginal microecology, especially in women with BV and mixed vaginitis. Data of *C. trachomatis* showed statistical differences and data of *M. genitalium* was close to statistical differences.

In previous studies, no precise data suggest *M. genitalium* is associated with specific organisms that can cause vaginitis, such as *Trichomonas vaginalis* or *Candida albicans*. In contrast, evidence of a relationship with BV is inconsistent, with some studies showing decreased risk of BV in women with *M. genitalium* [19, 20], one showing increased risk [21], and other showing no association [22–24]. A recent longitudinal study found that infection rate of *M. genitalium* might be higher in the presence of BV [25], and *C. trachomatis* showed similar situations. *M. genitalium* alone, however, does not appear to be a cause of vaginitis, which may explain why *M. genitalium* is not routinely screened.

Although the infection rates of *M. genitalium* in this research is not as high as expected, we still can't ignore the detriment of *M. genitalium* to female reproductive system. Some basic studies have confirmed the damage of *M. genitalium* to female reproductive health. *M. genitalium* was significantly associated with a 70% increase in risk of cervicitis [26]. Acute *M. genitalium* infection of endocervical cells causes microvilli destruction and persistent *M. genitalium* infection may lead to resultant chronic inflammation. In vitro and animal researches have observed microscopic evidence of ciliary damage of human fallopian tube infected with *M. genitalium*, albeit the damage was more moderate than that seen with *C. trachomatis* or *N. gonorrhoeae* infection. Cilia damage leads to potential damage to ectopic pregnancy and infertility [27]. In fact most women with infertility due to fallopian tube damage do not have a history of acute PID [28], which suggests that subclinical infections may be more common than acute PID and could equally cause reproductive harm [29]. The association between *M. genitalium* infection and tubal infertility has been demonstrated, independent of chlamydial infection [30]. Our study did not include women with PID or a history of PID, so the prevalence of *M. genitalium* in our data was not as high as expected. We are now recruiting women with PID and the epidemiological data of *M. genitalium* in Chinese women will be more complete in the near future.

Logistic regression analysis showed that alcoholism and abnormal vaginal microecology were positively correlated with *C. trachomatis* and *M. genitalium* infection, which confirmed the above results. We also observed that increasing age, being married and multiple births were negatively correlated with *C. trachomatis* infection. Perhaps being married and having children means that sexual partners are relatively stable and reproductive function have not been impaired. After all, there is a clear correlation between multiple sexual partners/diverse sexual styles and *C. trachomatis* infection.

Associations between *M. genitalium* and most female reproductive tract syndromes have been demonstrated although the statistical significance of results is not always uniform. Fairly consistent evidence shows that women infected with *M. genitalium* are at increased risk of PID, infertility, and adverse pregnancy outcomes and it is probably an important pathogen. However, *M. genitalium* is not a “new chlamydia” due to their different characteristics of infection, as well as the possible differences in pathogenesis. Additional data will be required before routine screening can be recommended for *M. genitalium*. But this research prefers that attention should be paid to *M. genitalium* infection in young women with abnormal vaginal microecology while having childbearing needs.

## Conclusion

We performed the current study to evaluated the prevalence and risk factors of *M. genitalium* and *C. trachomatis* in Chinese women with lower genital infection. Our results suggested that prevalence of *C. trachomatis* and *M. genitalium* were higher in women with abnormal vaginal microbiology especially in women with BV and mixed vaginitis. The overall prevalence of *M. genitalium* is lower than that of *C. trachomatis*, while they have similarities in the characteristics of infection. Although *M. genitalium* is not routinely screened as *C. trachomatis* in young women, attention should be paid to *M. genitalium* infection in young women with abnormal vaginal microecology or having childbearing needs. However, there are several limitations in this study which should be noted. First, the population sample size was still low compared to huge Chinese population. Second, it would be a limitation that we have not include female with PID and this part are still in research. Third, given the study population is highly selected, the prevalence rates given in this study may not be generalisable to the whole population. We are giving treatment and following up the infected women, hoping to know the average duration of *M. genitalium* in Chinese women and the changes of vaginal microbiology after clearance of pathogen. Hope for meaningful results in the future. At the same time, prospective studies evaluating whether screening programs and targeted treatment of *M. genitalium* improve reproductive outcomes in women are necessary to guide public health policy for this newly emerging pathogen.

## Supplementary Information

**Additional file 1:** **Table S1**. Value assignmentof incorporated factors in univariate and multivariate analysis.

## Data Availability

The datasets used and/or analysed during the current study available from the corresponding author on reasonable request.
