# Supplementary material for: Prevalence of Mycoplasma genitalium and Chlamydia trachomatis in Chinese female with lower reproductive tract infection: a multicenter epidemiological survey
Source: BMC Infect Dis. 2023 Jan 5;23:2. doi: 10.1186/s12879-022-07975-2 (PMC9814310; doi:10.1186/s12879-022-07975-2)
Supplement: Supplementary file 1 — Additional file 1: Table S1. Value assignmentof incorporated factors in univariate and multivariate analysis. [file 12879_2022_7975_MOESM1_ESM.docx]

Additional file 1

Table S1. Value assignment of incorporated factors in univariate and multivariate analysis

| Incorporated factors | Value assignment |
| --- | --- |
| Age (Years old) | 1. ＜20 2. [20-25) 3. [25-30) 4. [30-35) 5. [35-40) 6. ≥40 |
| Education level | 1. No degree 2. Bachelor's degree 3. Master degree or above |
| Being married | 1. No 2. Yes |
| Alcohol abuse | 1. No 2. Yes |
| Vaginal douching | 1. No 2. Occasionally 3. Often |
| Using sanitary pads freguently | 1. No 2. Occasionally 3. Often(over than 1/3 time every month) |
| Age of first sex (Years old) | 1. ＜15 2. [15-20) 3. ≥20 |
| Various style of sex | 1. vaginal intercourse 2. Various styles of sexual intercourse |
| Number of sex partner | 1. 1 2. ≥2 |
| Gravidity and parity | 1. 0 2. 1 3. 2 4. ≥3 |
| Number of abortion | 1. ≤1 2. ≥2 |
| Way of contraception | 1. Condom 2. Other methods |
| Vulva redness | 1. No 2. Yes 3. ulceration of vulva |
| Purulent discharge of cervix | 1. No 2. Yes |
| Vaginal cleanliness | 1. Degree I-II 2. Degree III |
| Vaginal microecology | 1. Normal 2. Abnormal |
